# Membrane mediated toppling mechanism of the folate energy coupling factor transporter

**DOI:** 10.1038/s41467-020-15554-9

**Published:** 2020-04-09

**Authors:** Ignacio Faustino, Haleh Abdizadeh, Paulo C. T. Souza, Aike Jeucken, Weronika K. Stanek, Albert Guskov, Dirk J. Slotboom, Siewert J. Marrink

**Affiliations:** University of Groningen, Groningen Biomolecular Sciences and Biotechnology Institute, Nijenborgh 4, 9747 AG Groningen, The Netherlands

**Keywords:** Biochemistry, Biophysics, Computational biology and bioinformatics, Structural biology

## Abstract

Energy coupling factor (ECF) transporters are responsible for the uptake of micronutrients in bacteria and archaea. They consist of an integral membrane unit, the S-component, and a tripartite ECF module. It has been proposed that the S-component mediates the substrate transport by toppling over in the membrane when docking onto an ECF module. Here, we present multi-scale molecular dynamics simulations and in vitro experiments to study the molecular toppling mechanism of the S-component of a folate-specific ECF transporter. Simulations reveal a strong bending of the membrane around the ECF module that provides a driving force for toppling of the S-component. The stability of the toppled state depends on the presence of non-bilayer forming lipids, as confirmed by folate transport activity measurements. Together, our data provide evidence for a lipid-dependent toppling-based mechanism for the folate-specific ECF transporter, a mechanism that might apply to other ECF transporters.

## Abstract (web summary)

Energy coupling factor (ECF) transporters are responsible for the uptake of micronutrients and consist of an integral membrane unit, the S-component, which confers substrate specificity. Here, authors present multi-scale molecular dynamics simulations and in vitro experiments to study the molecular toppling mechanism of the S-component of a folate-specific ECF transporter.

## Introduction

Energy coupling factor (ECF) transporters catalyze the uptake of micronutrients in prokaryotes^1–5^. These transporters are a class of ATP-binding cassette (ABC) transporters that comprise four subunits or components: two similar or identical cytoplasmic ATPases (EcfA and EcfA′) and two transmembrane proteins (EcfT or T-component, and EcfS or S-component)^1,2,4^. The integral membrane S-component binds the substrate and translocates it across the membrane by rotating or toppling in the bilayer. The driving force for this unusual toppling step is unknown. The rest of the ECF transporter, also called ECF module, is indirectly involved in the transport step by stabilizing the inward facing (toppled) state of the S-component, and catalyzing ATP hydrolysis to recover the “ready-to-bind” outward facing (canonical) state of the S-component^4,6–8^. ECF transporters are classified in two groups. In group I, the S-component and the ECF module are dedicated to form a single complex and they are usually encoded in the same gene cluster^2^. The ECF module of group II transporters can associate with multiple S-components allowing the transport of more than one substrate. The S-components of group II transporters can dissociate from the ECF module during the transport cycle. The genes encoding the S-components and ECF module of group II transporters are located at different positions in the bacterial genome^2^.

Several crystal structures of isolated S-components for different vitamins and transition metals have been solved in the substrate-bound state, and full ECF complexes in the substrate-free state^6,9–13^. The former structures show what seems to be the pre-translocation state, after the S-component binds its substrate and before associating with the ECF module. The latter reveals a post-translocation state with the substrate-free S-component in the toppled orientation with the empty binding site facing the cytoplasm^6–8,14,15^.

**Fig. 1:**
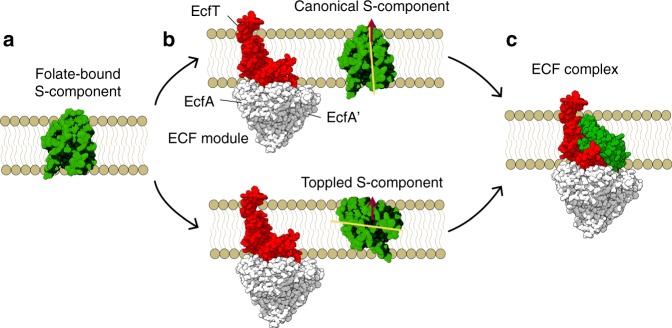
ECF-FolT2 complex structure and transport cycle. **a** The S-component in its folate-bound conformation, **b** the folate-bound S-component binds to the ECF module (consisting of the EcfA, EcfA′, and EcfT subunits) in two possible orientations, canonical (upper part), or toppled (lower part). The orientation of the S-component is represented by the yellow line. The brown arrow shows the direction of the bilayer normal, **c** the S-component binds the ECF module to form the ECF complex. The S-component is in green, EcfT is in red, and EcfA/EcfA′ is in white surface. The bilayer is shown schematically.

Based on these structures and other experimental data, a working model for the transport has been proposed that explains the different orientations of the S-component in the available crystallographic structures (Fig. 1)^6^. The toppling mechanism involves the rotation of the S-component in the lipid bilayer after binding the substrate, which moves the substrate from a location close to the extracellular side of the membrane to the cytoplasmic side. In the toppled state the S-component can dock onto the ECF module and release its substrate into the cytosol. A subsequent reverse rotation to the canonical state should occur to reset the S-component and allow other substrate-bound S-components to dock onto the ECF module. The binding and hydrolysis of ATP would trigger the reverse rotation, which is associated with release of the S-component from the ECF module in group II transporters. This step is presumably caused by a series of conformational changes in the ECF module that the S-component is attached to^6^.

To understand the toppling process at the molecular level, and to investigate a possible role of lipid composition, molecular dynamics (MD) simulations are a suitable tool^16–19^. In particular coarse-grained (CG) models have proven their efficiency to simulate inherently slow processes such as reorientation of membrane proteins and identification of protein–lipid-binding sites^20^. Here, we use CG MD simulations based on the Martini force field^21^ to study the toppling mechanism of the ECF transporter for folate. For this transporter, high-resolution structural information is available for both solitary S-component with the bound substrate folate, and for FolT2 in the *apo* form in complex with the ECF module^6^.

Our simulations reveal how different membrane compositions affect the preferred stability of the S-component in the toppled orientation, depending on the state of the protein (*holo* versus *apo*). Moreover, we observe how membrane bending facilitates the interaction between the ECF module and the S-component, providing a driving force for toppling. The predictions from the simulations are subsequently tested by an experimental assay in proteoliposomes, where activity of the reconstituted ECF-FolT2 transporter is measured as a function of lipid composition. The combined results suggest a lipid-mediated mechanism for substrate translocation across membranes.

## Results

### The toppled S-component is stabilized by non-bilayer lipids

**Fig. 2:**
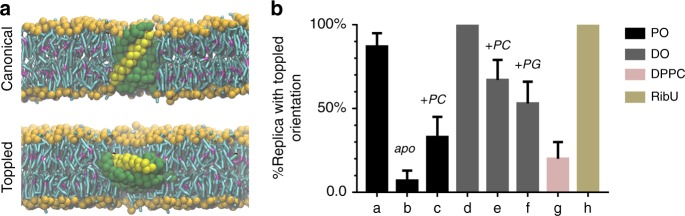
Orientation of the S-component. **a** Snapshots of the S-component in canonical and toppled orientations. The S-component is shown in green and helix 5 in yellow. Both are plotted in van der Waals CG surface representation. The phosphate groups and tails of the lipids are shown in orange and cyan, respectively, with magenta for unsaturated parts of the tails. **b** Bar graph of orientational preference of the S-component starting from toppled state in different lipid environments in either *apo* or *holo* form (a: POPE-POPG-CL-*holo* S-component (ratio: 70:25:5), b: POPE-POPG-CL-*apo* S-component (ratio: 70:25:5), c: POPE-POPG-POPC-*holo* S-component (ratio: 60:20:20), d: DOPE-DOPG-DOPC-*holo* S-component (ratio: 60:20:20), e: DOPE-DOPG-DOPC-*holo* S-component (ratio: 50:13:37), f: DOPE-DOPG-DOPC-*holo* S-component (ratio: 30:30:40), g: DPPC-*holo* S-component, h: POPE-POPG-CL-*holo* RibU S-component (ratio: 70:25:5)). The error bars represent standard deviation. The lipid abbreviations are according to Table 1. Source data are provided as a Source Data file.

We first studied the orientational preference of the folate-specific S-component using CG MD simulations. The protein was embedded in a bacterial membrane model composed of POPE (1-palmitoyl-2-oleoyl-sn-glycero-3-phosphoethanolamine), POPG (1-palmitoyl-2-oleoyl-sn-glycero-3-phosphoglycerol) and CL (cardiolipin) lipids (ratio 70:25:5, see Table 1). The choice of lipids was done to include the major components of the *E. coli* membranes, which are mostly PEs (phosphatidylethanolamines), PGs (phosphatidylglycerols), and CLs^22–24^. The S-component was considered either in its *apo* or *holo* conformational state, which differ primarily in the position of the loop L1 that shields off the substrate-binding site from the environment when the substrate is bound. The orientation of the protein was quantified according to the angle formed by the helix 5 of the S-component and the normal of the lipid bilayer (Fig. 2a). We performed 5 μs unrestrained MD simulations pre-orienting the S-component either in a canonical (helix 5 parallel to the bilayer normal) or in a toppled (helix 5 perpendicular to bilayer normal) orientation. For each condition 15 replicas were simulated. Our results show that when the S-component was initially oriented in the canonical state, the protein maintained such an orientation in all replicas regardless of whether it was in the *holo* or *apo* state (Supplementary Fig. 1).

**Table 1:** Summary of the unbiased MD simulations performed in this study.

Simulation	Duration	Lipid composition (ratios)
**Coarse grain**		
*apo* S-component (Canonical)	15 × 5 μs	POPE, POPG, CL (70:25:5)
*apo* S-component (Toppled)	15 × 5 μs	
*holo* S-component (Canonical)	15 × 5 μs	
*holo* S-component (Toppled)	15 × 5 μs	
*holo* S-component (Toppled)	15 × 5 μs	DOPE, DOPG, DOPC (50:37:13)
*holo* S-component (Toppled)	15 × 5 μs	DOPE, DOPG, DOPC (30:30:40)
*holo* S-component (Toppled)	15 × 5 μs	DOPE, DOPG, DOPC (60:20:20)
*holo* S-component (Toppled)	15 × 5 μs	POPE, POPG, POPC (60:20:20)
*holo* S-component (Toppled)	15 × 5 μs	DPPC
*holo* RibU S-component (Toppled)	15 × 5 μs	POPE, POPG, CL (70:25:5)
ECF transporter bound to S-component	1 × 32 μs	POPE, POPG, CL (70:25:5)
ECF transporter without S-component	1 × 32 μs	
ECF transporter without S-component	1 × 20 μs	DOPG, DOPE, DOPC (60:20:20)
ECF transporter without S-component	1 × 20 μs	POPE, POPG, POPC (60:20:20)
**All-atom**		
ECF transporter bound to S-component	1 × 100 ns	POPE, POPG, CL (70:25:5)
ECF transporter without S-component	1 × 100 ns	POPE, POPG, CL (70:25:5)

The lipid abbreviations are as follows:

*POPE* 1-palmitoyl-2-oleoyl-sn-glycero-3-phosphoethanolamine, *POPG* 1-palmitoyl-2-oleoyl sn-glycero-3-phosphoglycerol, *CL* cardiolipin, *DOPC* 1,2-dioleoyl-sn-glycero-3-phosphocholine, *DOPE* 1,2-dioleoyl-sn-glycero-3-phosphoethanolamine, *DOPG* 1,2-dioleoyl-sn-glycero-3-phosphoglycerol, *POPC,* 1-palmitoyl-2-oleoyl-sn-glycero-3-phosphocholine, *DPPC* 1,2-dipalmitoyl-sn-glycero-3-phosphocholine.

When the *holo* form of the S-component was initially toppled in the membrane, the S-component remained almost exclusively in the toppled orientation (Fig. 2b, a and Supplementary Fig. 1). In contrast, when placing the *apo* form in the toppled orientation, the S-component rotated back to the canonical orientation soon after the simulation had started in almost all replicas (Fig. 2b, b). Apparently, the toppled state is more stable for the *holo* form, and likely represents at least a metastable configuration when the substrate is bound. The presence of kinetic barriers between the toppled and canonical configurations could explain the lack of spontaneous toppling events on the microsecond time scale of our simulations.

Next, to test the effect of lipid environment on the orientational stability of the S-component, we repeated the simulations with the *holo* form in model membranes with different lipid compositions (Table 1). In particular, we tested the importance of changing headgroups (PC (phosphatidylcholine) versus PE) as well as lipid tails (increasing the level of unsaturation). The results on the stability of the toppled orientation are also presented in Fig. 2b (c–h). Two trends are visible: replacing PC with PE headgroups results in an increased stability of the toppled configuration. The same is true when replacing PO (palmitoyl-oleoyl) lipids by DO (dioleoyl) lipids. Together, these results point to an important role for non-bilayer forming lipids, i.e. lipids featuring a relatively small headgroup and/or bulky unsaturated tails, in stabilizing the toppled state. This is most noticeable in case of the lipid membrane enriched in DOPE (1,2-dioleoyl-sn-glycero-3-phosphoethanolamine) (Fig. 2b, d), for which the toppled orientation was stable in all 15 replicas. The increased stability could be due to a lowering of the free energy of this state or by increasing the barrier toward adopting the canonical state.

### Membrane distortion near the ECF module facilitates toppling

**Fig. 3:**
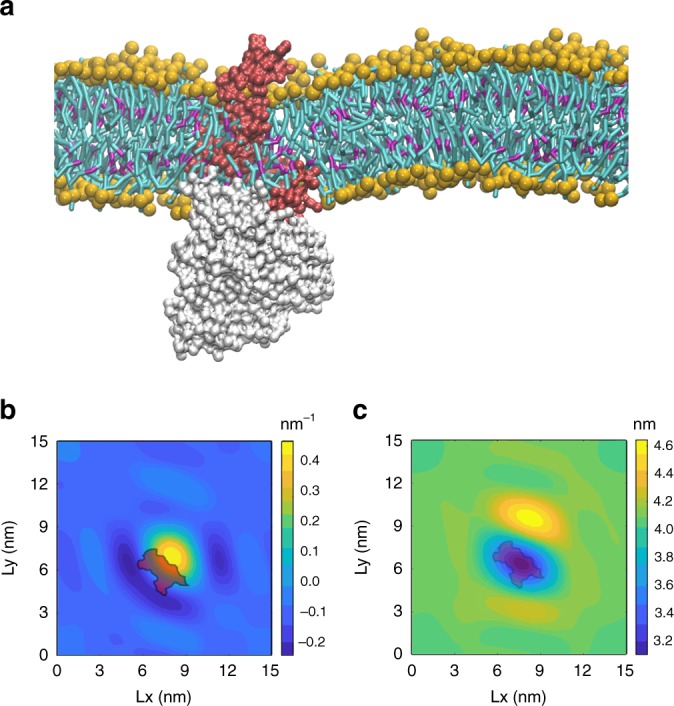
ECF-FolT2 induced membrane deformation. **a** MD snapshot showing the local membrane bending around the ECF module. EcfT is colored in red, EcfA and EcfA′ in white, and the phosphate groups of the lipids are colored in orange. Lipid tails are depicted in cyan with magenta for the unsaturated parts. **b** Curvature map around the ECF module (outline) for the upper leaflet, Lx and Ly denote bilayer lateral directions. **c** Membrane thickness map around the ECF module.

To study the possible role of the ECF module in the toppling process, we performed a 32 μs CG MD simulation of the ECF module in the absence of the S-component, and without bound ATP, in the bacterial model membrane composed of POPE, POPG, and CL lipids (ratio: 70:25:5, see Table 1). In the course of the simulation, we observed tilting of the ECF module inside the membrane and bending of the bilayer around the protein (Fig. 3a). We calculated the local curvature of a bilayer segment around the ECF module following a protocol previously published^25^. A negative curvature was imparted to the bilayer around the complex where the S-component can associate with the ECF module. Simultaneously, we observed a positive curvature at the other side of the ECF module (Fig. 3b). A similar curvature profile around the full four subunit ECF complex is also noticeable in previous self-assembly simulations based on the same CG model^26^. In addition to the curvature, we also analyzed the thickness of the bilayer patch by fitting a fourth degree polynomial to the positions of the phosphate groups separately for lipids in the upper and lower leaflets^27^. The average bilayer thickness is shown in Fig. 3c. While the thickness of the lipid bilayer in the bulk region is ~4 nm, we observed thinning of the bilayer around the protein in the S-component binding position to ~3.6 nm. To assess whether or not the observed curvature and thinning are results of the use of a CG model, we converted the final snapshot of the CG ECF system into an atomistic representation using the backwards protocol (see Methods for more details)^28^. After performing an MD simulation of 100 ns, we observed that the bending and local thinning were also maintained in the atomistic lipid bilayer (Supplementary Fig. 2). Apparently, the shape and surface properties of the ECF module are responsible for reshaping of the surrounding membrane. Additional simulations using different membrane compositions (Table 1) show very similar behavior, with the largest deformation observed in the presence of DOPE lipids (Supplementary Fig. 3).

Based on these results, we propose that the combination of a tilted protein, and a negatively curved and thin bilayer prepares a platform where the S-component can associate with the ECF module. Near such a platform, the S-component can use the curved bilayer to slide towards its ultimate docking position. As a result of the thin bilayer near the docking site, the S-component can tilt to avoid the exposure to the water. Therefore, the S-component could remain in the canonical orientation after binding its substrate without having to actively topple over. According to our proposed mechanism, the S-component could follow the curvature of the membrane during association to and dissociation from the ECF module. To study the interaction of the S-component with the ECF module in more detail, we repeated the simulations of the ECF module but now in complex with the S-component in its *apo* form, as found in the crystal structures.

**Fig. 4:**
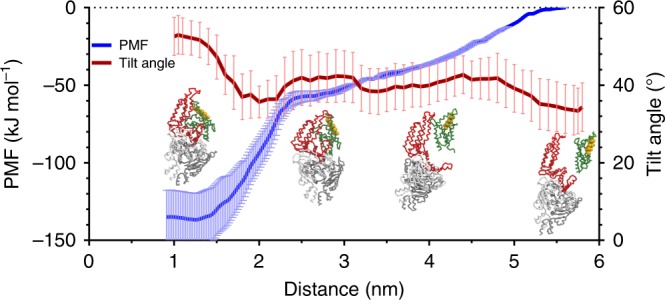
Dissociation of S-component from ECF complex. Potential of mean force of ECF module and S-component association (blue). Error bars were computed with bootstrapping. Average tilt angle between helix 5 and the positive *z*-direction of the simulation box along the dissociation pathway (red). Error bars are standard deviations. Representative snapshots of ECF and S-component along the dissociation pathway are shown. Toppled state is on the left and canonical state is on the right. Color coding is the same as Fig. 1. Helix 5 is highlighted in yellow. Source data are provided as a Source Data file.

After 32 μs of unbiased MD simulation, however, the complex remains stable with no spontaneous unbinding of the S-component (Supplementary Fig. 4). As the unbinding of the S-component is likely an ATP activated process in reality, we opted for using biased umbrella sampling simulations to trigger the dissociation of the S-component from the ECF module and estimate the free energy profile of the process. The resulting free energy profile and representative snapshots are shown in Fig. 4, together with data showing the temporal evolution of the tilt angle of the S-component during unbinding. The snapshots show that the S-component loses its binding mode to the ECF complex completely at ~2.5 nm distance (measured between the centers of mass of EcfT and the bottom of the S-component, see Methods). With respect to the crystallographic binding mode, the dissociation free energy at this point is around ~80 kJ mol^−1^. Interestingly, the free energy profile beyond this distance does not plateau, but keeps gradually increasing for almost another 3 nm, even though significant direct protein–protein contacts are no longer observed. This effect is correlated with the long-range membrane bending induced by the ECF complex in the bilayer (cf. Fig. 3a). Thus, an additional binding free energy of 55 kJ mol^−1^ can be attributed to the bilayer curvature effect. During the unbinding process, the tilt angle gradually decreases from around 50° in the crystallographic binding mode to values around 30° as observed for the isolated *apo* S-component in the canonical orientation. Although the presented free energy profile is somewhat biased (see Methods), it supports the notion of a membrane assisted toppling of the S-component when interacting with the ECF module.

Thus, our results suggest that the membrane deformation around the ECF module may assist in reorienting the S-component toward a toppled state.

To provide more detail on the role of individual lipids in shaping the membrane around the ECF module, we performed a computational lipid fingerprint analysis. To this end, we quantified the relative depletion and enrichment of lipids in the annular shell of the protein by means of a depletion–enrichment (D–E) index, according to an earlier protocol^29^. The results (Supplementary Table 1) indicate a preferred interaction of CL and POPG lipids with the ECF transporter with distinct binding sites, for both lipids around the protein (Supplementary Figs. 5 and  6).

### Experiments confirm lipid dependency of transport efficiency

**Fig. 5:**
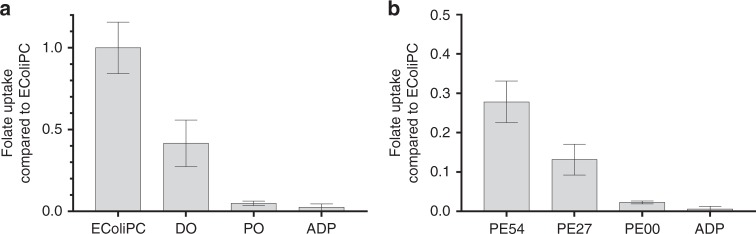
Folate uptake activity depends on lipid composition. Folate uptake by ECF-FolT2 reconstituted in proteoliposomes was measured with liposomes made of different phospholipids and normalized to activity observed in *E. coli* polar lipids mixed with egg yolk PC (3:1 w/w) (*E. coli* PC). **a** Effect of tail composition with similar headgroup distribution. DO refers to only DO lipids, and PO to only PO lipids. **b** Effect of headgroup (PE) composition with same tail composition (DO). PE54, PE27, and PE00, contain 54%, 27%, and 0% PE, respectively. ADP indicated a negative control in which proteoliposomes with *E. coli* PC lipid composition were loaded with ADP instead of ATP. Exact lipid compositions can be found in Table 2. Error bars are standard deviations. Source data are provided as a Source Data file.

Our simulations reveal a potential important role for lipids in the transport process of folate by ECF-FolT2. In particular, we predict that non-bilayer forming lipids are able to stabilize the toppling of the S-component (Fig. 2), an essential step in the proposed transport mechanism. Besides, our data suggest that anionic lipids may play a role as well. To test the effect of lipid composition on transport efficiency, folate uptake was measured using purified ECF-FolT2 reconstituted in liposomes of varying compositions of synthetic lipids^6^. Phospholipids with either DO or PO acyl chains were used, the abundance of lipids with headgroups PE, PG, and PC was varied systematically, and CL was either present or absent. All transport rates were benchmarked against the rates observed in proteoliposomes consisting of *E. coli* polar lipids mixed with egg yolk PC. The latter gave rise to higher transport activity than any of the synthetic lipid mixtures tested, indicating that a complex lipid mixture in terms of headgroups and acyl chains is beneficial for ECF-FolT2 functioning. Nonetheless, activities up to 40% of the benchmark could be obtained in synthetic lipid mixtures, allowing the assessment of the dependence on specific lipids (Fig. 5). Protein levels in the proteoliposomes were comparable in all experiments, see supporting Supplementary Fig. 7. Our analysis revealed a strong dependence of the transport activity on acyl chain identity. Robust transport activity was observed in liposomes containing DO lipids as long as lipids with PE headgroups were present, whereas liposomes consisting exclusively of PO lipids did not support transport. We propose that the lower activity in liposomes with PO lipids could be due to less bending of the lipid bilayer (Supplementary Fig. 3B). In liposomes consisting of DO lipids, there was a strong dependence of the transport activity on the fraction of PE lipids, with activity completely abolished in the absence thereof. Cardiolipin was not essential for transport activity and did not significantly alter transport activity (*p* = 0.1623, Welch two-sample *t*-test, Supplementary Fig. 8a). Variation of the amount of the negatively charged lipid, PG, also did not significantly alter the transport rates (Supplementary Fig. 8b).

**Table 2:** Summary of the lipid ratios in experiments.

	*E. coli* PC	DO	PO	ADP	PE54	PE27	PE00	PG00	PG12.5	PG25	PG37.5	DO Plus_CL
PC	25	27	27	25	27	40	54	37.5	25	12.5	0	25
PE	50	54	54	50	54	27	0	62.5	62.5	62.5	62.5	50
PG	17	19	19	17	19	33	46	0	12.5	25	37.5	17
CL	7.5	0	0	7.5	0	0	0	0	0	0	0	8
Acyl chain composition	Mix	DO 18:1, 18:1	PO 16:0, 18:1	Mix	DO 18:1, 18:1	DO 18:1, 18:1	DO 18:1, 18:1	DO 18:1, 18:1	DO 18:1, 18:1	DO 18:1, 18:1	DO 18:1, 18:1	DO 18:1, 18:1

## Discussion

Based on the results we obtained and the data published in previous work on ECF transporters, we propose a mechanism for the transport of folate that builds on the one proposed by Swier et al.^6^. Like Swier et al. and others^7^, we have assumed that the substrate-free conformation of the S-component would initially align in the canonical orientation. This is supported by earlier experiments performed with the riboflavin-specific ECF transporter RibU^30^. However, the S-components in all available structures of full ECF transporters are oriented in the toppled state and interact with EcfT through hydrophobic interactions^6–8,14^.

**Fig. 6:**
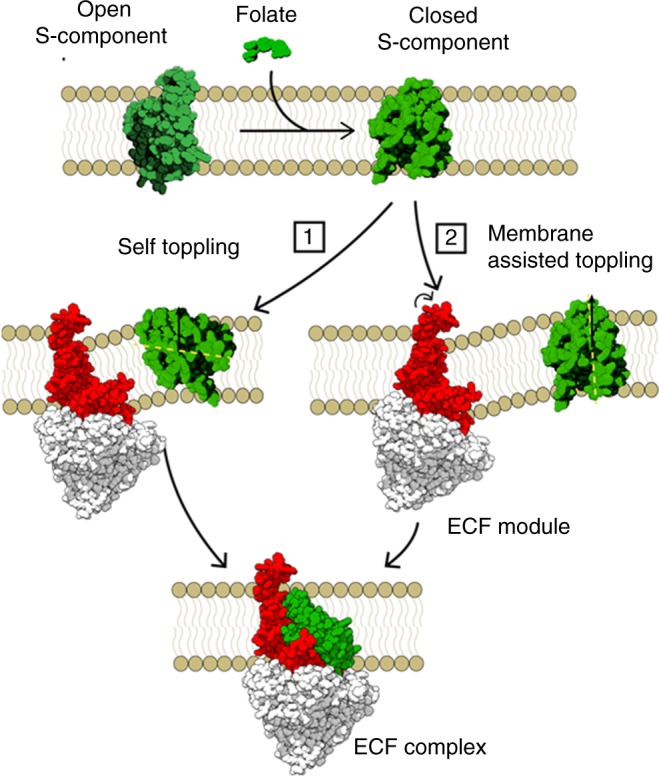
Mechanism proposed for the transport of folate by the group II ECF transporter ECF-FolT2. Once the open (canonical) S-component binds folate there are two pathways that the substrate-bound S-component can follow to bind the hydrophobic surface of the ECF module. In the first, the S-component can topple over inside the membrane possibly due to local fluctuations of the membrane and bind the ECF module in the orientation observed in crystallographic structures of ECF complexes. As a second pathway, the S-component can slide towards the ECF module thanks to the negative bending around the ECF module.

Upon substrate binding, we propose that the docking to the ECF module could occur in two ways (Fig. 6). The first possible mechanism involves the spontaneous toppling of the *holo* state inside the membrane. Such option is supported by our MD simulations with the *holo* state of the S-component. Our results, however, contradict previous results obtained by Josts et al. using MD simulations with the thiamine-bound S-component of YkoE transporter and other group II S-components including another substrate-bound S-component^11^. According to Josts et al., all investigated S-components went from the initial toppled to the canonical orientation within 1 μs simulation. One important difference is the lipid composition used in those simulations. Josts et al. used a lipid bilayer with DPPC (1,2-dipalmitoyl-sn-glycero-3-phosphocholine) lipids with different membrane properties than our lipid composition (POPE: POPG: CL), which is similar to what is found in *E. coli* lipid extracts. Specifically, we showed that PE lipids, as well as an increase in the number of oleoyl tails, are important to stabilize the toppled state. To ascertain that the discrepancy is due to the lipid composition, we performed additional MD simulations with the *holo* form of the S-component in pure DPPC membranes. As expected, the simulations show a destabilization of the toppled orientation favoring the canonical orientation, in 15 replicas (Fig. 2b, g).

In order to evaluate the general applicability of the stability of the closed state in the toppled orientation, we also performed MD simulations with the S-component of another group II ECF transporter^12,13^. Although S-components show specificity for different ligands, they all share the same global fold^6,12,13,30^. We performed 15 replicas of 5 µs MD simulations with the *holo* form of the S-component of the riboflavin transporter RibU^9^ using the same POPE:POPG:CL lipid composition we have previously used. The results obtained using the pre-oriented toppled S-component of RibU showed stability in this orientation in all the replicas (Fig. 2b, h). These results suggest that the application of the toppling mechanism can be extended to other group II ECF transporters.

The second mechanism that arises from the simulations of the ECF module shows membrane distortion around the ECF module in the pre-docking state that could facilitate the association with the S-component (Fig. 6, pathway 2). The coupling domain of EcfT provides a hydrophobic platform that binds the surrounding lipid tails generating a local protein-induced curvature in the membrane that would facilitate the association with the S-component without requiring a complete toppling over in the lipid bilayer. Our CG MD simulations, which are supported by atomistic simulations, show how the EcfT induces a local distortion that could guide the S-component association to the ECF module. Simulation of the direct docking process (placing the S-component in its *holo* form at a distance away from the ECF module) was not successful, likely hampered by kinetic barriers involving lipid desolvation from the binding interface. The dissociation of the S-component, however, could be captured using biased umbrella sampling simulations. These simulations corrobarated a possible role of the membrane curvature in providing a driving force for reorientation of the S-component. In vivo, the initial destabilization of the binding interface could be triggered by the binding of ATP and the resulting disruption of the hydrophobic interactions between EcfT and the S-component.

Several studies have pointed out to the allosteric modulation of membrane proteins functionality upon binding of anionic lipids. These lipids can influence membrane protein activity, complex formation and localization of proteins into specific phases in membranes^31–34^. Our simulations show that anionic lipids, and in particular CLs, migrate to specific locations on the membrane exposed surface of ECF-FolT2. Our experiments do not show any obvious dependence of the transport efficiency of ECF-FolT2 on CLs, or other anionic lipids, and therefore their specific role, if any, remains unclear. These results are in line with the notion that CL binding sites are commonly found on a large variety of membrane proteins, but their specific roles are often not known^16^.

In conclusion, our simulations and experimental data not only show that the functioning of the ECF transporter ECF-FolT2 is strongly affected by the lipid composition of the membrane, we also propose a specific mechanism by which protein-induced bilayer deformation can provide a driving force for the unusual toppling mechanism utilized by ECF transporters. Membrane bending and thinning around the ECF module may allow for toppling of the S-component relative to the ECF module, while remaining largely un-toppled relative to the lipid bilayer surface. In this way kinetic barriers which could prevent efficient toppling in the membrane may be overcome.

## Methods

### Coarse-grained MD simulations

We used the crystal structure of folate ECF transporter since the structures of the *apo* and *holo* forms were recently published. The crystallographic structures of the folate-bound protein (PDB code: 5D0Y) and the *apo* form of the ECF transporter containing the four subunits (PDB code: 5JSZ) were embedded in lipid bilayers with different compositions (Table 1). ATP and folate were not included in the CG models. Control simulations with the S-component of the RibU ECF transporter (PDB code: 3P5N) were also performed. The ECF module was positioned in the membrane according to the data derived from the OPM database^33^. The orientation of the principal *z*-axis of the protein was set to be parallel to the normal of the lipid bilayer. For all unbiased simulations, we employed the non-polarizable Martini 2.2 model^21,35–37^. The systems were hydrated using the standard water model in Martini, including 10% antifreeze type water beads^21^. A physiological concentration of 150 mM of NaCl was also included in the systems to match physiological conditions. The systems were first simulated for 4 ns allowing the membrane and the solvent to adapt around the proteins, which were kept fixed using position restraints. The restraints were subsequently removed and the system was equilibrated for another 4 ns. We then run 15 replicas of 5 µs for each system except for the simulations with the ECF complex (see Table 1). We used the Verlet cut-off scheme according to the GROMACS definition^38^. Temperatures of the protein, the lipids and the solvent were kept at 303 K with the stochastic velocity rescaling thermostat^39^. For the pressure, we used the semi-isotropic coupling at 1 bar using the Parrinello-Rahman barostat^40^. All simulations were performed with GROMACS (version 2016.1)^38^.

### Atomistic MD simulations

These simulations used the CHARMM36 force field^41^ in the NpT ensemble, with the temperature coupled weakly to a heat bath at 303 K, using the velocity rescale thermostat^39^ with a coupling time of 0.1 ps, and coupled to a reference pressure of 1.0 bar, using a Parrinello−Rahman barostat^40^ with a coupling time of 4.0 ps and a compressibility of 4.5 × 10^−5^ bar^−1^. Electrostatic interactions were calculated using PME (Particle Mesh Ewald)^42,43^, with a real-space cut-off of 1.2 nm. Van der Waals interactions were switched to zero between 1.2 and 1.4 nm. Neighbor lists were updated every 10 steps. Bonds involving hydrogens were constrained using the LINCS algorithm^44^. Water was modeled explicitly using the CHARMM three-point transferable intermolecular potential (TIP3P) model. The integration time step used was 2 fs and the overall center of mass motion was removed every 10 steps. Topologies for CHARMM36 were generated according to the implementation in GROMACS.

### Free energy calculation

As protein–protein interactions in Martini 2 are usually overestimated^45,46^, we resorted to the recently reparametrized version of the Martini force field, called Martini 3^47,48^, to compute the unbinding free energy profile of the S-component from the ECF complex. CG protein models were generated using the new version of the program Martinize^36,49^. Elastic networks were applied to each monomer of the ECF complex, with a distance cut-off of 0.85 nm using a force constant of 1300 kJ mol^−1^ nm^−2^. To further increase stability of the native protein folds during the unbinding process, additional harmonic bonds were added between three pair of residues of the ECF complex: Gln96 (chain A)–Leu213 (chain D), Ser172 (chain A)–Gly175 (chain B) and Gly110 (chain B)–Arg179 (chain D). They mimic hydrogen bonds between backbone amide groups of neighboring monomers. According to preliminary simulations in a POPE, POPG, CL (70:25:5) bilayer, the behavior of ECF and S-component (isolated and in complex) are similar to what was already observed in simulations with Martini 2 and reported in the main manuscript. The free energy profile was computed as a potential of mean force (PMF) using Umbrella Sampling^50^, and the free energies were extracted by the weighted histogram analysis method (WHAM)^51^, as implemented by Grossfield^52^. The reaction coordinate was the distance between the centers of mass of EcfT and the bottom of the S-component (defined as residues 69–75 and 127–133). A total of 51 windows with the restraining distances spaced 0.1 nm apart were used, with a simple harmonic umbrella potential using a force constant of 2500 kJ mol^−1^ nm^−2^. Minimum sampling time for each window was 20 µs. It is worth to notice that the obtained free energy only reflects one of the components of the binding affinity of the S-component to ECF, as the biased simulations performed do not fully include all possible conformational changes or protein–protein interfaces. It also reflects the specific pathway hypothesized based on the bilayer curvature around the ECF complex.

### Analysis

All the analysis was performed using GROMACS^38^, VMD^53^ and local scripts. Structures were visualized using VMD. To quantify the orientational stability of the toppled configuration, we calculated the percentage of time the S-component stays in the toppled orientation in simulations starting from a toppled S-component in 15 replicas. If the S-component stays in the toppled state for more than 50% of the simulation time, we count that replica as stable toppled orientation. The toppled orientation is stable if the angle between the helix 5 and the normal of the membrane is between 75° and 125° (Supplementary Fig. 1). We calculated the standard deviation based on a binomial distribution. The tilt angle analysis was performed using the GROMACS tool *gmx gangle*.

To identify the lipid binding sites, we computed the densities of the lipids at the protein surface using the GROMACS density tool. To characterize the CL and POPG-binding sites in a greater detail we used the following protocol. For each frame of the 32 µs simulation of the ECF complex we defined a list of residues simultaneously in contact with a unique CL or POPG lipid. A contact was counted when a residue was within 0.7 nm of the lipid headgroup (phosphatidyl and glycerol CG beads). The D–E index of a given lipid type was calculated for 0.7 nm as the distance cut-off from the protein^29^. For each lipid type *L*, we first defined the ratio of lipid *L* within the given cut-off to the total number of lipids (ratio(*L*)_*x*_). Then, the ratio of the lipid *L* with respect to bulk was calculated (ratio(*L*)_bulk_). The D–E index was calculated from the following ratio:

1 $${\mathrm{{D-E}}} = \frac{{{\mathrm{{ratio}}}\left( L \right)_x}}{{{\mathrm{{ratio}}}\left( L \right)_{\mathrm{{bulk}}}}}.$$

### Protein expression and purification

ECF-FolT2 from *Lactococcus Delbrueckii* was expressed in *E. coli* MC1061 cells (ATCC# 53338, CGSC# 6649)^54^ from the plasmid pBAD HisECF FolT2StrepII as described by Swier et al.^6^. Briefly, cells were cultivated in LB medium at 37 °C until an OD of around 0.6 and protein expression was induced with arabinose (final concentration of 0.01% w/v) for 3 h. Subsequently, cells were harvested (10 min, 7460*g*, 4 °C) and washed once with 50 mM KPi, pH 7.5. Cells were resuspended in 50 mM KPi, pH 7.5, 200 μM PMSF, 1 mM MgSO_4_, supplemented with ~50 μg DNase per ml and lysed using a Constant Cell Disruption System (Constant Systems Ltd, UK) by 1 passage at 25 kPsi at 4 °C. Cell debris removed was by centrifugation (30 min, 38,400*g*, 4 °C) and membrane fractions were collected by ultra-centrifugation (180 min, 186,000*g*, 4 °C). These membrane fractions were resuspended in 50 mM KPi, pH 7.5 and frozen in liquid nitrogen and stored at −80 °C until used.

Membranes were solubilized using 1% n-dodecyl-β-d-maltopyranoside (DDM, Anatrace) in 50 mM KPi, pH 7.5, 300 mM NaCl for 1 h at 4 °C. Non-solubilized material was removed by ultra-centrifugation (30 min, 287,000*g*, 4 °C), and the supernatant was incubated with buffer-equilibrated Ni^2+^-sepharose resin for 60 min. This mixture was transferred to a 10 ml disposable column (Bio-Rad) and drained, followed by a 20-column volume wash with wash buffer (50 mM KPi, pH 7.5, 300 mM NaCl, 50 mM Imidazole, 0.05% (w/v) DDM). Bound protein was eluted using three-step elution of 300, 750, and 500 μl of elution buffer (50 mM KPi, pH 7.5, 300 mM NaCl, 500 mM Imidazole, 0.05% (w/v) DDM). The second elution fraction was spun down on a tabletop centrifuge for 5 min at 13,000 rpm, 4 °C before injection on a Superdex 200 10/300 gel filtration column (GE Healthcare) which was pre-equilibrated with gel filtration buffer (50 mM KPi, pH 7.5, 150 mM NaCl, 0.05% (w/v) DDM). The fractions containing ECF-FolT2 were pulled together and directly used for reconstitution.

### Reconstitution into proteoliposomes

Lipids dissolved in chloroform were mixed in different ratios and dried under a stream of nitrogen gas. The obtained lipid film was dissolved in 50 mM KPi, pH 7.5 to a concentration of 20 mg ml^−1^ followed by passing 11× through a 400 nm polycarbonate filter (Avestin) using an extruder. The liposomes were destabilized by adding triton X-100 until half of the initial absorbance at 540 nm was reached. Freshly purified ECF-FolT2 was mixed in the ratio 1:300 protein:lipid (w/w) with the destabilized liposomes and left for 30 min incubation at room temperature. Thirty-two milligrams of Biobeads-SM2 per 1 ml of protein–liposome suspension were added and incubated for 30 min at room temperature. Nineteen milligrams of Biobeads-SM2 per 1 ml of protein–liposome suspension and incubation was continued at 4 °C for 60 min followed by another addition of 24 mg of Biobeads-SM2 per 1 ml of protein–liposome suspension and the suspension was incubated overnight. A final addition of 36 mg of Biobeads-SM2 per 1 ml of protein–liposome suspension was incubated for 60 min and proteoliposomes were collected (45 min, 287,000*g*, 4 °C) and aliquots were flash-frozen using liquid nitrogen.

### Radiolabeled folate uptake assay

Frozen proteoliposomes were thawed and MgATP or MgADP was added to a final concentration of 5 mM. Three cycles of freeze–thawing were performed and the suspension was passed 11 times through a 400 nm polycarbonate filter (Avestin). Afterwards proteoliposomes were collected (45 min, 287,000*g*, 4 °C) and resuspended in 50 mM KPi, pH 7.5 to a final protein concentration of 0.4 μg protein per μl.

For each timepoint in the uptake assay, liposomes containing 1 μg of ECF-FolT2 were added to 200 μl 50 mM KPi, pH 7.5 containing 100 nM folate (95 nM “cold” folate and 5 nM [3, 5, 7, 9-3 H] radiolabeled folate (American Radiolabeled Chemicals). The reaction was incubated for 0, 1, 2, 3, 4 min at 30 °C while stirring. At these time points 2 ml ice-cold 50 mM KPi, pH 7.5 was added and the solution was filtered over a BA-85 nitrocellulose filter, followed by one wash of 2 ml ice-cold 50 mM KPi, pH 7.5 of the filter. Filters were dissolved in 1.8 ml Filter count Scintillation liquid (PerkinElmer) and radioactivity was measured on a PerkinElmer Tri-Carb 2800 TR isotope counter. A linear regression was performed per uptake curve and normalized to the activity in *E. coli* polar lipids mixed with egg yolk PC (3:1 w/w).

### Reporting summary

Further information on research design is available in the Nature Research Reporting Summary linked to this article.

## Supplementary information

Supplementary Information

Reporting Summary

## Data Availability

Data supporting the findings of this manuscript are available from the corresponding authors upon request. A reporting summary for this Article is available as a Supplementary Information file. The source data underlying Figs. 2b, 4, 5, Supplementary Fig. 7 and Supplementary Fig. 8 are provided as Supplementary Information.

## Source Data

Source Data
