# Supplementary material for: Membrane mediated toppling mechanism of the folate energy coupling factor transporter
Source: Nat Commun. 2020 Apr 9;11:1763. doi: 10.1038/s41467-020-15554-9 (PMC7145868; doi:10.1038/s41467-020-15554-9)
Supplement: Supplementary file 1 — Supplementary Information [file 41467_2020_15554_MOESM1_ESM.pdf]

SUPPORTING INFORMATION

Membrane mediated toppling mechanism of the folate energy coupling factor transporter

Faustino and Abdizadeh et al.

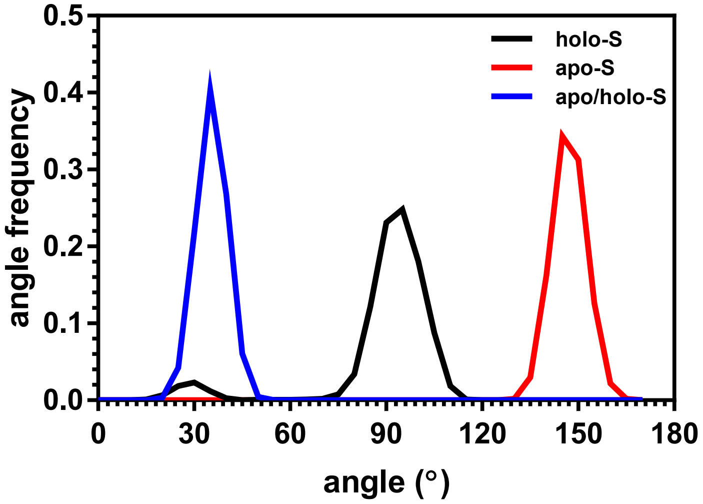

Supplementary Figure 1. Distribution of the angle formed between the helix 5 and bilayer normal. Simulations with the S-component initially in the toppled state in POPE, POPG, CL (ratio: 70:25:5) are shown in black and red. Simulations with the S-component initially in the canonical state are shown in blue. Angles around 30° or 150° represent a canonical orientation of the S-component, and an angle around 90° represents a toppled, orientation.

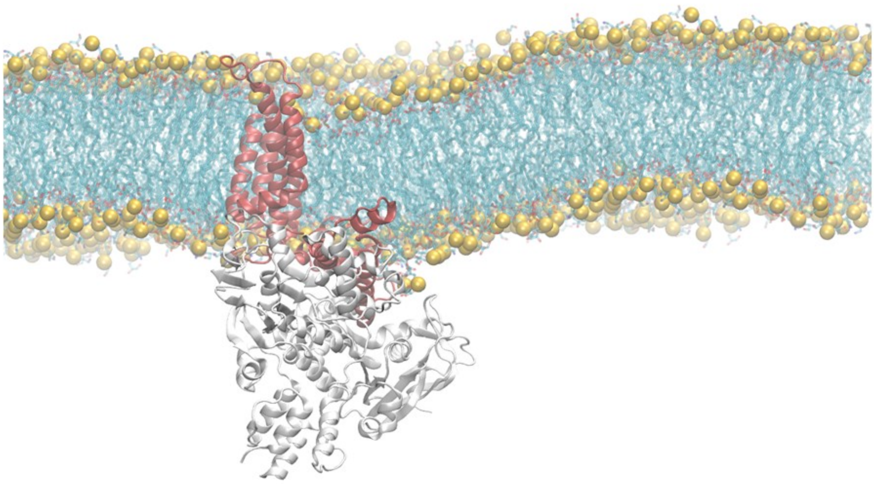

Supplementary Figure 2. Snapshot of membrane deformation around ECF module in POPE, POPG, CL (ratio: 70:25:5) lipid composition at atomistic detail. Color codes are the same as Fig. 3A.

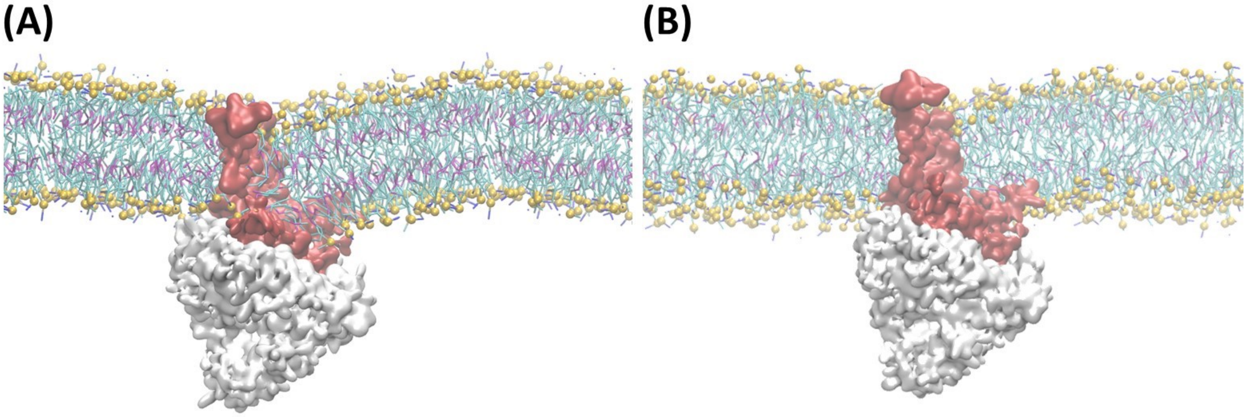

Supplementary Figure 3. Membrane deformation around ECF module: (A) DOPE, DOPG, DOPC (ratio: 60:20:20), (B) POPE, POPG, POPC (ratio: 60:20:20) lipid compositions at CG level. Color codes are the same as Fig. 3A.

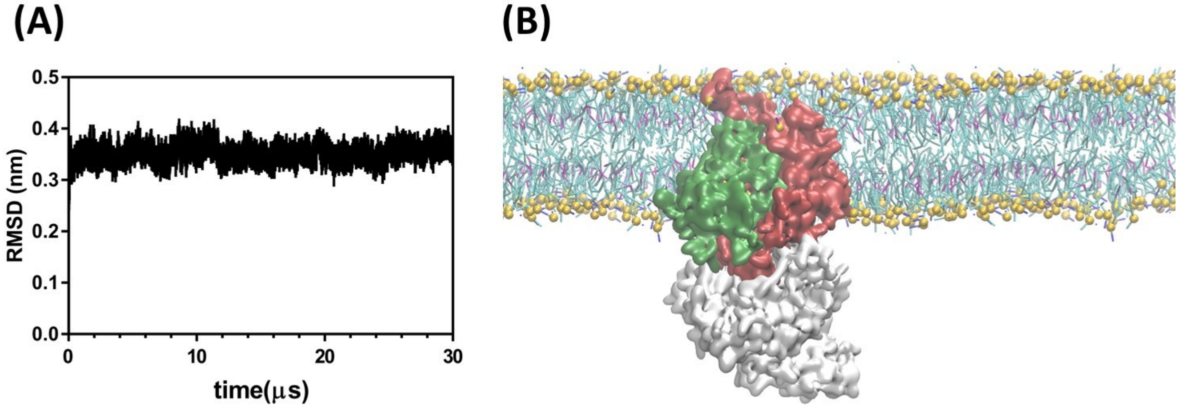

Supplementary Figure 4. (A) RMSD for ECF complex in POPE, POPG, CL (ratio:70:25:5) at CG level. The overall fold of the protein complex in the lipid bilayer remains similar to the initial configuration (after minimization and equilibration) with backbone RMSD of 0.35 ± 0.02 (SD) nm. (B) Snapshot for ECF complex in POPE, POPG, CL (ratio:70:25:5).

Supplementary Discussion

Prediction of anionic lipid enrichment around the ECF module

Based on the 32 μs long simulation of ECF complex system in POPE:POPG:CL (ratio: 75:20:5), we characterize the distinctive nature of the lipid environment surrounding the protein, using a distance cutoff criterion of 0.7 nm. We then compare the composition of this lipid shell around the protein to the bulk membrane composition, expressed as the relative depletion–enrichment (D–E) index for different categories of lipids. This is done to provide more detail on the role of individual lipids in shaping the membrane around the ECF complex and ECF module. If the value of the D-E index is greater than 1, there is enrichment of the specific lipid type around the protein whereas an index value lower than 1 indicates that the mean density of the lipids in the bulk region is higher compared to the density of the lipids close to the protein. Supplementary Table 1 indicates the D-E index for the ECF in complex with the S-component and without the S-component.

Supplementary Table 1. Lipid environments (D-E index) of ECF transporter

	Upper	Lower	Upper	Lower	Upper	Lower
	POPE		POPG		CL	
ECF complex	0.6	0.9	0.8	1.8	1.8	2.8
ECF module	0.6	0.9	0.7	1.8	1.6	2.6

These results indicate a preferred interaction of CL and lower leaflet POPG lipids with the ECF proteins. Further analysis reveals the presence of distinct binding sites, both for CL and PG lipids around the protein (Supplementary Figure 5 and Supplementary Figure 6).

Lipid binding sites

In order to study the interactions between the protein and lipid species, we first quantified the mean number of lipids in the proximity of the protein based on the number of phosphate head groups within a distance cutoff of 0.7 nm (Supplementary Figure 5). We measured 24 POPE, 13 POPG and 8 CL lipids around the ECF complex (including the S-component), and 24 POPE, 13 POPG and 9 CL lipids around the ECF module in contact with the protein in both leaflets during the 32 μs of the simulation. There is a higher number of lipids around the protein in the lower leaflet of the membrane, as expected from the wider shape of the transporter in the cytosolic side. To better interpret the lipid population around the protein, we calculated the time-averaged lipid densities for each lipid type. Supplementary Figure 5 shows the density maps calculated for both the ECF complex and the ECF module alone over 32 μs. CL molecules establish a notable contact with the protein in both systems, and in both lower and upper leaflets. The high density of CL lipids designates that while POPE lipids are the most abundant lipid types in the simulations, lipid exchange occurs due to the favorable interactions established by CL lipids with the proteins. We also observed a high density of POPG molecules around the protein in the lower leaflet. In fact, calculations show that a positive electrostatic potential surface exists on the transmembrane part of the proteins which triggers the migration of anionic CL and POPG lipids from the bulk to the protein surface. This creates an acidic annulus around the protein, which may have an unknown role for ECF transporter function.

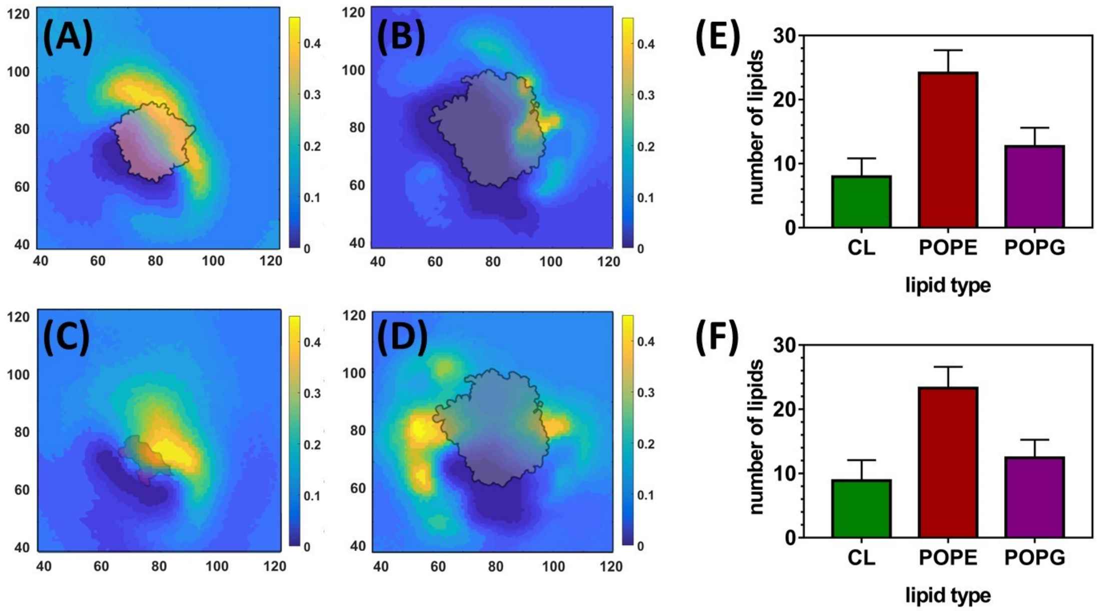

Supplementary Figure 5. Density maps of CL lipids. (A) Density maps of CL lipids around the ECF complex (outline) in the upper and (B) lower leaflets. (C) Density maps of CL lipids around the ECF module (outline) in the upper and (D) lower leaflets. (E) Number of lipids around the ECF complex and (F) the ECF module.

For the ECF complex and ECF module, we calculated the number of CL and POPG head groups within 0.7 nm of the proteins and identified binding sites for CL and POPG lipids on the S-component and on the EcfT (Supplementary Figure 6). Here, we report protein residues that were in contact with a unique CL or POPG head group for more than 0.05 of the simulation time. CL lipids were found in the region corresponding to resides Ser9 - Leu11 (site 1), Phe55 - Phe61 (site 2), Tyr101 - Gln103, Ile107 – Arg109 (site 3) and Ser131 - Met139 (site 4) on the S-component. The CL binding sites found on the transmembrane region of EcfT (in either ECF complex or ECF module) correspond to residues Ile73 – Met75 (site 5), Tyr93 - His95 (site 6), Lys150 – Met155 (site 7) and Thr262 - Lys264 (site 8).

We find that POPG binding sites on the S-component are Ser9 - Leu11 (site 1'), Ala37 - Val41 (site 2') , Tyr101 - Gln103 and Ile107 - Arg109 (site 3'), Asn137 - Met139 (site 4'), and Arg145 – Glu149 (site 5'). The POPG binding sites on EcfT (in either ECF complex or ECF module) are Gly7 – Tyr9 (site 6'), Pro71 – Met75 (site 7') and Lys195 – Lys198 (site 8').

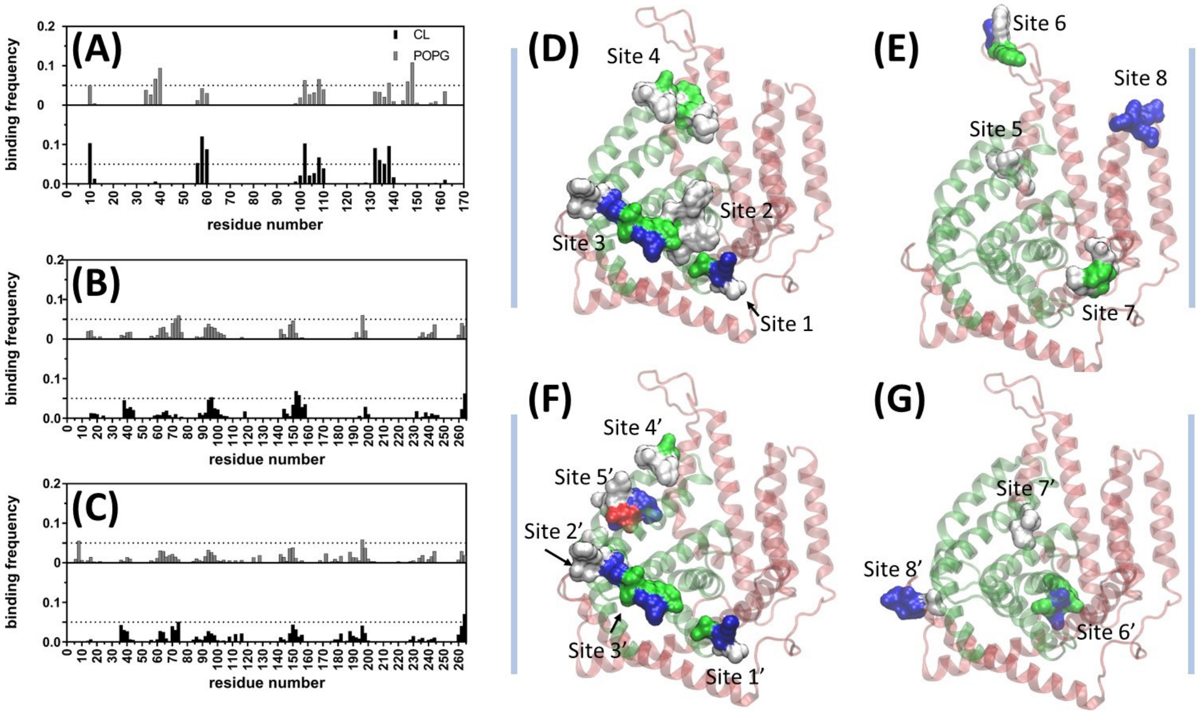

Supplementary Figure 6. Lipid binding sites. Frequency of CL and POPG lipids binding to the protein per residue number. (A) S-component in the ECF complex system, (B) EcfT in ECF complex system (C) EcfT in ECF module system. (D) Side representation of the CL binding sites for the S-component and (E) Side representation of the CL binding sites for EcfT. (F) Side representation of the POPG binding sites for the S-component and (G) Side representation of the POPG binding sites for EcfT. S-component is in green and EcfT in red. Residues involved in lipid binding are represented in van der Waals and colored according to their type: non-polar (white), basic (blue), acidic (red) and polar (green). Membrane is displayed in blue bars.

To confirm the presence of the CL and POPG binding sites, we performed atomistic MD simulations from the final configuration in the CG simulations of both ECF complex and ECF module. The 100 ns simulation following the transformation confirmed the stability of CL and POPG binding sites.

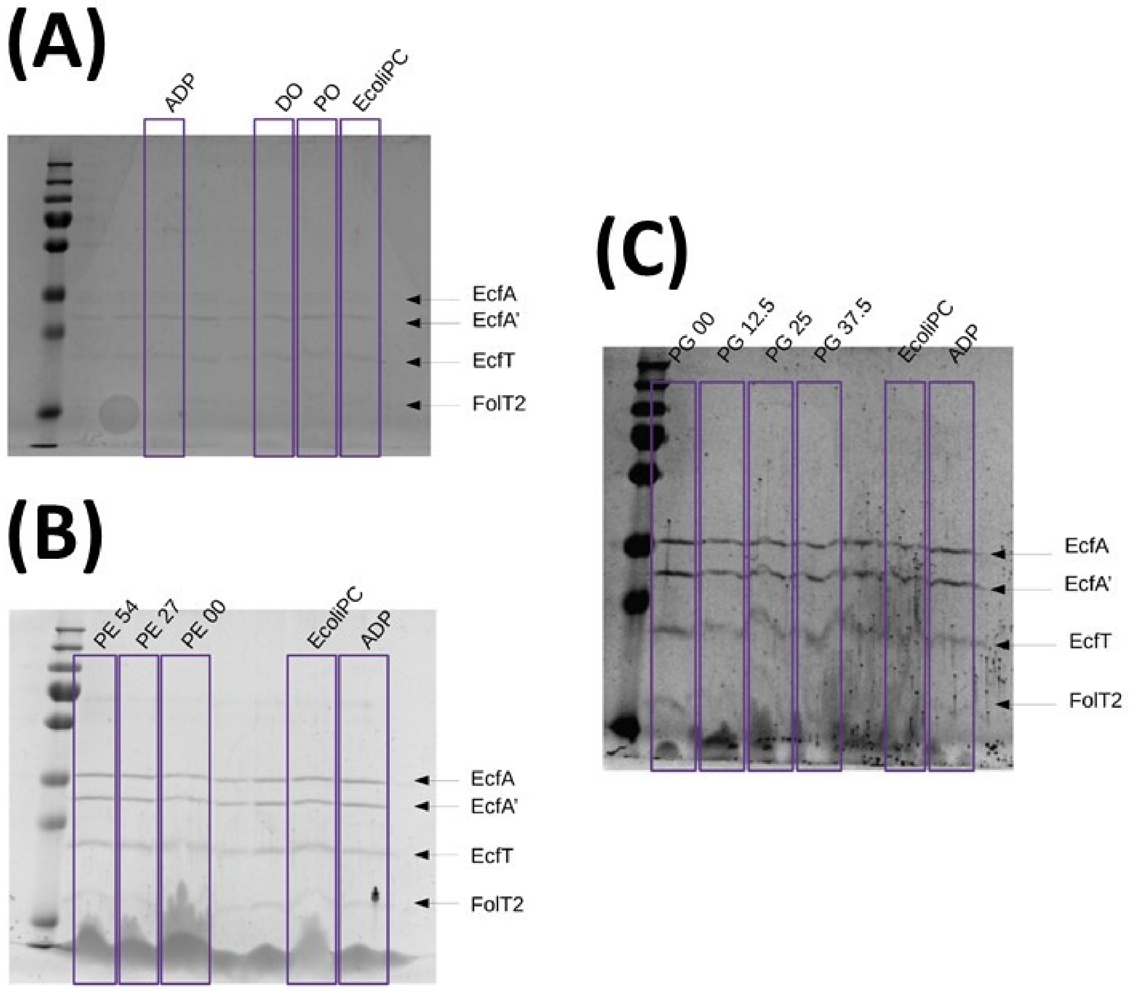

Supplementary Figure 7. Coomassie stained sds-page gel. The gels show the proteoliposomes as used for the uptake experiments. It depicts similar levels of protein among the proteoliposomes of different lipid composition. Panel A corresponds to Fig. 5A, panel B to Fig. 5B, and panel C corresponds to Supplementary Figure 8. Source data are provided as a Source Data file.

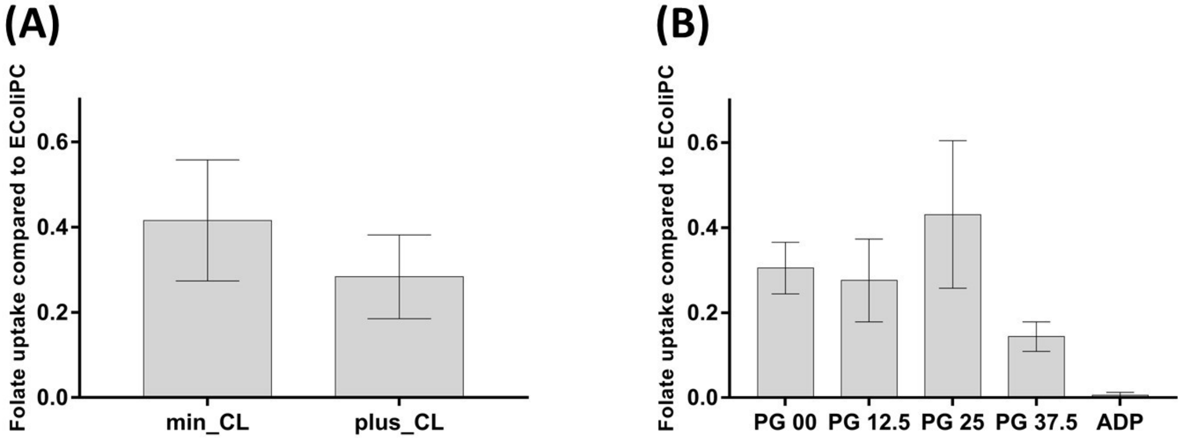

Supplementary Figure 8. Folate uptake activity in liposomes in the absence or presence of CL and with different concentrations of PG. (A) Uptake activity of ECF-FolT2 was measured in liposomes in the absence (min_CL) or presence (plus_CL) of 8 % CL. (B) Uptake activity of ECF-FolT2 was measured in liposomes with different concentrations of PG (0, 12.5, 25, 37.5 %) in DO lipids, called PG 00, PG 12.5, PG 25, PG 37.5, respectively. ADP refers to proteoliposomes with *EcoliPC* lipids where ADP is encapsulated instead of ATP. Table 2 summarizes the exact ratios of used lipids. Source data are provided as a Source Data file.
