# Supplementary material for: Membrane mediated toppling mechanism of the folate energy coupling factor transporter
Source: Nat Commun. 2020 Apr 9;11:1763. doi: 10.1038/s41467-020-15554-9 (PMC7145868; doi:10.1038/s41467-020-15554-9)
Supplement: Supplementary file 2 — Reporting Summary [file 41467_2020_15554_MOESM2_ESM.pdf]

nature research

Corresponding author(s): Haleh Abdizadeh and Siewert-Jan Marrink

Last updated by author(s): Mar 5, 2020

Reporting Summary

Nature Research wishes to improve the reproducibility of the work that we publish. This form provides structure for consistency and transparency in reporting. For further information on Nature Research policies, see Authors & Referees and the Editorial Policy Checklist .

Statistics

For all statistical analyses, confirm that the following items are present in the figure legend, table legend, main text, or Methods section.

n/a	Confirmed	
☐	☒	The exact sample size (*n*) for each experimental group/condition, given as a discrete number and unit of measurement
☐	☒	A statement on whether measurements were taken from distinct samples or whether the same sample was measured repeatedly
☐	☒	The statistical test(s) used AND whether they are one- or two-sided<br>*Only common tests should be described solely by name; describe more complex techniques in the Methods section.*
☒	☐	A description of all covariates tested
☒	☐	A description of any assumptions or corrections, such as tests of normality and adjustment for multiple comparisons
☐	☒	A full description of the statistical parameters including central tendency (e.g. means) or other basic estimates (e.g. regression coefficient) AND variation (e.g. standard deviation) or associated estimates of uncertainty (e.g. confidence intervals)
☒	☐	For null hypothesis testing, the test statistic (e.g. *F*, *t*, *r*) with confidence intervals, effect sizes, degrees of freedom and *P* value noted<br>*Give P values as exact values whenever suitable.*
☒	☐	For Bayesian analysis, information on the choice of priors and Markov chain Monte Carlo settings
☒	☐	For hierarchical and complex designs, identification of the appropriate level for tests and full reporting of outcomes
☒	☐	Estimates of effect sizes (e.g. Cohen's *d*, Pearson's *r*), indicating how they were calculated

Our web collection on statistics for biologists contains articles on many of the points above.

Software and code

Policy information about availability of computer code

Data collection	To collect Molecular Dynamics trajectories, we used open source Gromacs version 2016.1 (www.gromacs.org). Raw data converted to plots using GraphPad Prism 7 (available upon request).
Data analysis	Standard Gromacs and Visual molecular dynamics (VMD) (https://www.ks.uiuc.edu/Research/vmd/) were used for data analysis. Analysis algorithms of thickness and curvature were programmed in C++ and obtained through personal communication with W. Pezeshkian as cited in the main text (Pezeshkian, W., Nåbo, L. J. & Ipsen, J. H. Cholera toxin B subunit induces local curvature on lipid bilayers. FEBS Open Bio 7, 1638–1645 (2017)). Analysis algorithm of depletion- enrichment index were programmed in Python, using MDAnalysis (https://www.mdanalysis.org) and NumPy packages. VMD was used to create images.

For manuscripts utilizing custom algorithms or software that are central to the research but not yet described in published literature, software must be made available to editors/reviewers. We strongly encourage code deposition in a community repository (e.g. GitHub). See the Nature Research guidelines for submitting code & software for further information.

Data

Policy information about availability of data

All manuscripts must include a data availability statement. This statement should provide the following information, where applicable:

- Accession codes, unique identifiers, or web links for publicly available datasets
- A list of figures that have associated raw data
- A description of any restrictions on data availability

Atomic coordinates have been deposited in the PDB under accession code 5jsz and 5d0y. All relevant data are available from the corresponding authors on request.

Field-specific reporting

Please select the one below that is the best fit for your research. If you are not sure, read the appropriate sections before making your selection.

- [x] Life sciences
- [ ] Behavioural & social sciences
- [ ] Ecological, evolutionary & environmental sciences

For a reference copy of the document with all sections, see nature.com/documents/nr-reporting-summary-flat.pdf

Life sciences study design

All studies must disclose on these points even when the disclosure is negative.

Sample size	Sample sizes were determined based on the author's experience of what is necessary to generate a convincing and compelling result.
Data exclusions	No data were excluded from the analyses in the simulations.
Replication	Simulation runs were repeated 15 times for S-component and membrane system. The replicates were initiated from separately assembled protein-membrane systems.
Randomization	*Describe how samples/organisms/participants were allocated into experimental groups. If allocation was not random, describe how covariates were controlled OR if this is not relevant to your study, explain why.*
Blinding	*Describe whether the investigators were blinded to group allocation during data collection and/or analysis. If blinding was not possible, describe why OR explain why blinding was not relevant to your study.*

Reporting for specific materials, systems and methods

We require information from authors about some types of materials, experimental systems and methods used in many studies. Here, indicate whether each material, system or method listed is relevant to your study. If you are not sure if a list item applies to your research, read the appropriate section before selecting a response.

Materials & experimental systems

n/a	Involved in the study
☒	☐ Antibodies
☒	☐ Eukaryotic cell lines
☒	☐ Palaeontology
☒	☐ Animals and other organisms
☒	☐ Human research participants
☒	☐ Clinical data

Methods

n/a	Involved in the study
☒	☐ ChIP-seq
☒	☐ Flow cytometry
☒	☐ MRI-based neuroimaging
